# Risk Factors for Mortality in Abdominal Infection Patients in ICU: A Retrospective Study From 2011 to 2018

**DOI:** 10.3389/fmed.2022.839284

**Published:** 2022-02-25

**Authors:** Xingzheng Luo, Lulan Li, Shuhua Ou, Zhenhua Zeng, Zhongqing Chen

**Affiliations:** ^1^Department of Critical Care Medicine, Affiliated Xiaolan Hospital, Southern Medical University (Xiaolan People's Hospital), Zhongshan, China; ^2^Department of Critical Care Medicine, Nanfang Hospital, The First School of Clinical Medicine, Southern Medical University, Guangzhou, China; ^3^Department of Infection, Affiliated Xiaolan Hospital, Southern Medical University (Xiaolan People's Hospital), Zhongshan, China

**Keywords:** abdominal infection, risk factors, prognosis, ICU, mortality

## Abstract

To identify the risk factors related to the patient's 28-day mortality, we retrospectively reviewed the records of patients with intra-abdominal infections admitted to the ICU of Nanfang Hospital, Southern Medical University from 2011 to 2018. Multivariate Cox proportional hazard regression analysis was used to identify independent risk factors for mortality. Four hundred and thirty-one patients with intra-abdominal infections were analyzed in the study. The 28-day mortality stepwise increased with greater severity of disease expression: 3.5% in infected patients without sepsis, 7.6% in septic patients, and 30.9% in patients with septic shock (*p* < 0.001). In multivariate analysis, independent risk factors for 28-day mortality were underlying chronic diseases (adjusted HR 3.137, 95% CI 1.425–6.906), high Sequential Organ Failure Assessment (SOFA) score (adjusted HR 1.285, 95% CI 1.160–1.424), low hematocrit (adjusted HR 1.099, 95% CI 1.042–1.161), and receiving more fluid within 72 h (adjusted HR 1.028, 95% CI 1.015–1.041). Compared to the first and last 4 years, the early use of antibiotics, the optimization of IAT strategies, and the restriction of positive fluid balance were related to the decline in mortality of IAIs in the later period. Therefore, underlying chronic diseases, high SOFA score, low hematocrit, and receiving more fluid within 72 h after ICU admission were independent risk factors for patients' poor prognosis.

## Introduction

Intra-abdominal infections (IAIs) is a common infectious disease in ICU (1, 2) with a high morbidity and mortality (3, 4). Compared with other infections, IAIs are more likely associated with septic shock and acute kidney injury (1, 5). Patients in ICU often have various underlying diseases. There are many factors, such as age, nutritional status, chronic underlying diseases, sepsis, organ failure, surgical intervention or infection removal and antibiotic therapy, may all play an important role in identifying patients' prognosis and assessing severity of diseases timely (3, 6–11). With the development of surgery or drainage methods, and adjustment in bundle treatment of sepsis and antibiotic treatment strategies (12), the abovementioned factors might not be applicable to the present conditions to predict mortality rate. Treatment of ICU-IAIs is very complicated and most IAIs patients have poor prognosis. Early identification of specific clinical feature and potential risk factors that may improve their survival is vital to clinicians, however, the existing literature is rarely reported about it.

To this end, we conducted a retrospective clinical study and analyzed the data of ICU-IAIs patients during the past 8 years in ICU of Nanfang Hospital, Southern Medical University to figure out the clinical characteristics and explore the relevant risk factors of 28-day mortality. Furthermore, we compared the differences in mortality between the previous 4 years and the next 4 years.

## Methods

We retrospectively analyzed the data of patients diagnosed with IAIs who were admitted to ICU of Nanfang Hospital, Southern Medical University from January 1, 2011 to December 31, 2018. We searched for relative information on the database of Nanfang Hospital from January 1, 2011 to December 31, 2018. The search was limited to “abdominal infection” or “peritonitis” or “intestinal fistula” or “anastomotic fistula” or “digestive tract perforation” and patients who have been admitted to ICU. The inclusion criteria for ICU-IAIs patients were listed as follows: (1) Age older than 18 years; (2) Meeting the criteria for IAI according to the 2005 International Sepsis Forum Consensus Conference (13). Patients who stayed in ICU for <24 h were excluded. By reviewing and collecting the relative data in the case system and tracking for the 28-day mortality, we figured out potential risk factors that would predict for the prognosis of the patients.

The research protocol was approved by the Ethical Committee of Nanfang Hospital, Southern Medical University (No.: NFEC-2019-162), and all research work was in compliance with the Declaration of Helsinki and the rules of Nanfang Hospital of Southern Medical University on clinical research.

## Definitions

The diagnostic criteria of IAIs are based on the definition in the 2005 International Sepsis Forum Consensus Conference (13). Those criteria are clinical manifestations (including abdominal pain and systemic inflammatory response syndromes such as fever, tachycardia, and shortness of breath) that match the signs and symptoms of IAI and the laboratory examination of peritoneal specimens meets the criteria for infection or IAI confirmed by surgery or microbiologic culture of peritoneal specimens. According to the Surgical Infection Society's (SIS) definition (14, 15), healthcare or hospital-associated IAIs (HA-IAIs) are defined as follows: patients who have been hospitalized for at least 48 h during the previous 90 days; patients residing in a skilled nursing or long-term care facility during the previous 30 days; patients who have received intravenous infusion therapy, wound care, or renal replacement therapy within the preceding 30 days; patients who have received several days of broad-spectrum antimicrobial therapy within the previous 90 days; patients who have post-operative infections; and patients known to have been colonized by or previously infected with a resistant pathogen. Patients not meeting those criteria were classified as CA-IAIs.

Underlying chronic diseases include the following conditions: chronic obstructive pulmonary disease; chronic heart failure (NYHA grade III-IV); metastatic cancer (metastasis confirmed by surgery or imaging methods); hematological malignancies (lymphoma, acute leukemia or multiple myeloma); liver cirrhosis; chronic renal failure (chronic renal insufficiency requires maintenance hemodialysis or combined with serum creatinine level > 300 μmol/L); immunosuppressive status (receiving corticosteroids within the past 6 months: Prednisolone equivalent ≥0.3 mg/kg per day for at least 1 month, severe malnutrition, congenital humoral or cellular immune deficiency); chemotherapy/radiotherapy status (received treatment within the past 6 months); human immunodeficiency virus (Human Immunodeficiency Virus, HIV) infection (HIV positive combined with clinical complications such as pneumocystis elvis pneumonia, Kaposi's sarcoma, lymphoma, tuberculosis or toxoplasmosis, etc.); diabetes.

Septic shock was regarded as patients with sepsis who needed vasopressor drugs to maintain the mean arterial pressure higher than 65 mmHg as well as the serum lactate level over 2 mmol/L after fluid resuscitation (16).

Initial antibiotic therapy (IAT) failure was defined as the initial antibiotic treatment cannot completely cover the organisms isolated from the intra-abdominal specimen.

### Data Collection

We collected the following baseline data: 1. Demographic data (age, gender); 2. Disease characteristics and underlying disease status: infection type, infection site, peritonitis type, Mannheim Peritonitis Index (MPI), Acute Physiology and Chronic Health Evaluation-II (APACHE-II), Sequential Organ Failure Assessment (SOFA) scores, various underlying chronic diseases and use of broad-spectrum antibiotics within 90 days or cortisone use; 3. Surgical data: timing of surgery or drainage, number of operations; 4. Laboratory test results; 5. Fluid balance, duration of mechanical ventilation and use of vasopressor drugs; 6. Microbiological culture results of intra-abdominal specimens (limited to abdominal effusion or pus or tissues obtained during surgery and effusion or drainage from the abdominal cavity within 24 h after ICU admission) and antimicrobial sensitivity test data; 7. Antibiotics treatment. When culture and susceptibility were obtained, we adjusted the antibiotics based on the susceptibility results. Unless otherwise specified, all variables were the results collected at the timepoint of ICU admission.

### Statistical Analysis

Stata/MP 15.0 software was used to establish a database and carry out statistical analysis. The patients were divided into two groups based on their 28-day survival status. Descriptive statistics were performed on continuous variables and categorical variables. Continuous variables conforming to the normal distribution are represented by *x* ± s, and continuous variables that are not normally distributed are represented by M (P25, P75). Categorical variables were expressed by frequency and percentage. Taking the time of admitted to ICU as the starting point, the primary endpoint was 28-day mortality, and the secondary endpoints were ICU mortality and hospital mortality. Kaplan-Meier survival analysis method was used to analyze mortality rate. Univariate COX regression was used to screen factors associated with 28-day mortality and calculated hazard ratio (HR) and 95% confidence interval (CI). Potential risk factors (*P* < 0.10) derived from the univariate analysis were further incorporated into the multivariate COX proportional hazard regression. The COX model used a backward stepwise to assess independent predictors associated with death, and the proportional hazard hypothesis test was performed.

Further analysis was to explore whether the mortality rate of patients in the study group has changed over time. The continuous variables were divided into two groups, the first 4 years and the next 4 years, respectively. Independent sample *t*-test or analysis of variance or Mann-Whitney *U*-test was used according to the situation. The comparison of categorical variables between groups was performed by χ2 test or Fisher's exact test. All tests used two-sided tests, and *P* < 0.05 was considered statistically significant.

## Results

**Figure 1:**
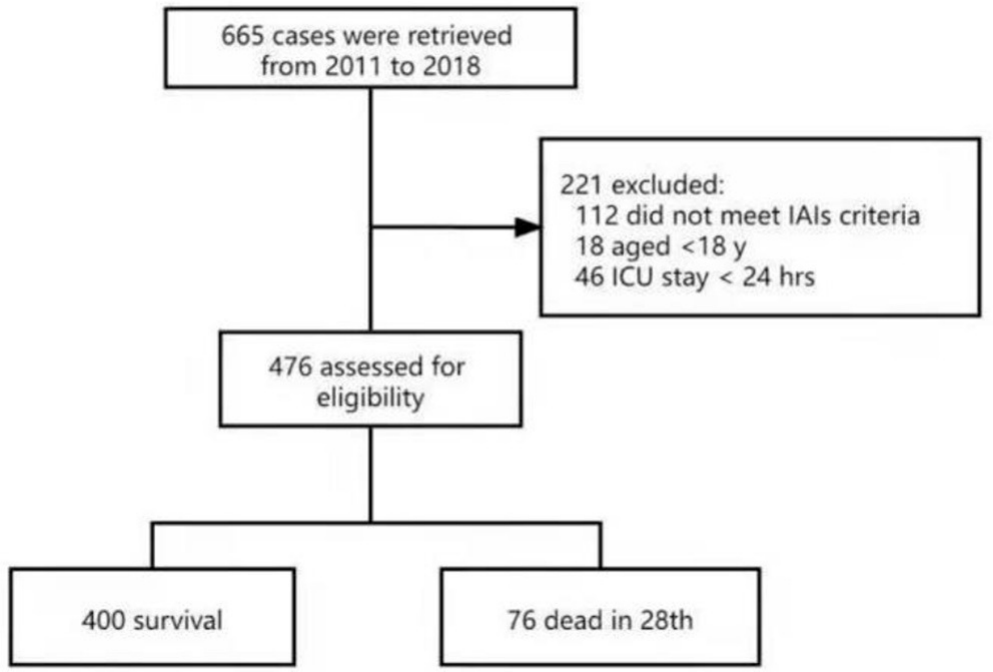
Flowchart of the study cohort.

There were 665 patients eligible for the present study; 112 patients did not meet the criteria of IAIs, 13 patients were peritonitis without IAIs and 18 were younger than 18 years old and 46 patients were hospitalized in ICU for <24 h. Finally, the remaining 476 patients were entered for analysis (Figure 1).

### Patient Characteristics

The median age of 476 patients was 60.5 (47, 71) years, including 319 (67.0%) males and 157 (33.0%) females. CA-IAIs and HA-IAIs were 209 (43.9%) and 267 (56.1%), respectively. The median length of stay in ICU was 4 days (interquartile range [2,9], full range [1,114]), and the median total hospital stay was 20 days (interquartile range [12,35], full range [2,155]). The median APACHE-II and SOFA were 15 [11, 20] and 5 [3, 7], respectively. When admitted to ICU, a total of 40.1% (191/476) patients had septic shock, and 60.9% (290/476) patients had acute kidney injury of varying degrees. Most of patients were completed peritoneal specimen culture, and a total of 527 organisms were isolated. *Escherichia coli, Klebsiella pneumoniae, Enterococcus faecium, Candida albican*s, and *Enterococcus faecalis* were the five most common organisms. The isolation rates of Enterococcus, non-fermenting bacteria and fungi were 27.6% (119/431), 11.4% (49/431), and 21.8% (94/431), respectively.

Fifty-two percent (247/476) of patients had underlying chronic diseases. The most common chronic diseases were metastatic cancer (21.6%), diabetes (12.8%), and immunosuppressive status (10.1%). ICU mortality, 28-day mortality, and overall hospital mortality were 12.4% (59/476), 16.0% (76/476), and 16.4% (78/476), respectively. The 28-day mortality stepwise increased with greater severity of disease expression: 3.5% (4/114) in infected patients without sepsis, 7.6% (13/171) in septic patients, and 30.9% (59/191) in patients with septic shock (*p* < 0.001).

Table 1 showed the results of the univariate COX analysis of the baseline characteristics of patients who died or survived at 28 days. Compared with patients in the survival group, the non-survival group had more HA-IAIs and postoperative IAIs (pIAIs), and showed higher APACHE-II score, SOFA score and MPI score. In addition, patients in non-survival group were mostly associated with fever or hypothermia, shortness of breath, SIRS, and septic shock, as well as a higher proportion of underlying chronic diseases.

**Table 1:** Demographic variables, setting of acquisition, baseline underlying condition, severity of condition, and clinical presentation of IAIs patients in ICU.

	**Total**	**Survivors**	**Non-survivors**	**Hazard ratio**	**95% CI**	***p*-values**
	***N* = 476**	***N* = 400**	***N* = 76**			
**Baseline characteristics**						
Age, year (median, IQR)	60.5 (47.0, 71.0)	60.0 (46.5–70.5)	61.0 (49.5–71.0)	1.010	0.996–1.024	0.158
Sex, Male *n* (%)	319 (67.0)	264 (66.0)	55 (72.4)	0.733	0.443–1.212	0.226
Setting of acquisition				2.550	1.500–4.336	<0.001
Community-acquired	209 (43.9)	191 (47.8)	18 (23.7)			
Health-care-associated/						
Hospital-acquired	267 (56.1)	209 (52.3)	58 (76.3)			
Postoperative IAIs, *n* (%)	127 (26.7)	93 (23.5)	34 (44.7)	2.107	1.339–3.314	0.001
Apache-II score (median, IQR)	15.0 (11.0, 20.0)	14.0 (10.0, 19.0)	23.0 (17.5, 28.0)	1.116	1.088–1.145	<0.001
SOFA score (median, IQR)	5.0 (3.0, 7.0)	4.0 (3.0–6.0)	8.5 (6.0–11.0)	1.272	1.203–1.344	<0.001
MPI (median, IQR)	25.0 (20.0, 31.0)	23.0 (19.0–30.0)	29.5 (24.5–32.0)	1.091	1.056–1.128	<0.001
Underlaying chronic disease, *n* (%)	247 (51.9)	183 (45.8)	64 (84.2)	5.732	3.018–10.887	<0.001
COPD	26 (5.5)	18 (4.5)	8 (10.5)			
Chronic heart failure	32 (6.7)	19 (4.8)	13 (17.1)			
Metastatic cancer	103 (21.6)	82 (20.5)	21 (27.6)			
Hematologic malignancy	10 (2.1)	6 (1.5)	4 (5.3)			
Cirrhosis	35 (7.4)	22 (5.5)	13 (17.1)			
Chronic renal failure	18 (3.8)	11 (2.8)	7 (9.2)			
Immunosuppression	48 (10.1)	29 (7.3)	19 (25.0)			
AIDS	1 (0.2)	0	1 (1.3)			
Diabetes mellitus	61 (12.8)	47 (11.8)	14 (18.4)			
Recent antibiotic therapy	125 (26.3)	100 (25.0)	25 (32.9)	1.326	0.820–2.142	0.250
**Clinical presentation**						
Fever or hypothermia^*^, *n* (%)	257 (54.0)	203 (50.8)	54 (71.1)	2.192	1.331–3.612	0.002
Tachypnea^#^, *n* (%)	265 (55.7)	214 (53.5)	51 (67.1)	1.589	0.982–2.571	0.060
SBP (median, IQR), mmHg	120.0 (105.0, 135.0)	120.5 (108.0, 136.5)	109.0 (91.0, 127.5)	0.976	0.966–0.985	<0.001
GCS sore (median, IQR)	15.0 (15.0, 15.0)	15.0 (15.0, 15.0)	15.0 (13.0, 15.0)	0.698	0.622–0.782	<0.001
qSOFA (median, IQR)	1.0 (0.0, 1.0)	1.0 (0.0, 1.0)	1.0 (1.0, 2.0)	2.298	1.774–2.975	<0.001
SIRS sore (median, IQR)	3.0 (2.0, 3.0)	3.0 (2.0, 3.0)	3.0 (2.5, 4.0)	1.539	1.208–1.960	<0.001
Acute kidney injury, *n* (%)	290 (60.9)	228 (57.0)	62 (81.6)	2.651	1.479–4.751	0.001
Septic shock, *n* (%)	191 (40.1)	132 (33.0)	59 (77.6)	5.687	3.312–9.767	<0.001

*IAIs, Intra-Abdominal Infections; ICU, Intensive Care Unit; CI, Confidence Interval; IQR, Interquartile Range; CA, Community-acquired; HA, healthcare or hospital-associated; APACHE-II, Acute Physiology and Chronic Health Evaluation-II; SOFA, Sequential Organ Failure Assessment; MPI, Mannheim Peritonitis Index; COPD, Chronic Obstructive Pulmonary Disease; AIDS, Acquired Immune Deficiency Syndrome; SBP, Systolic Blood Pressure; GCS, Glasgow Coma Scale; qSOFA, quick SOFA; SIRS, Systemic Inflammatory Response Syndrome*.

* *T ≥ 38°C or T ≤ 36°C*.

# *Breath rath ≥ 22/min*.

In terms of laboratory test results, compared with the survival group, the non-survival group had significantly higher serum PCT, lactate, creatinine and urea nitrogen levels, while the hematocrit and platelet counts were significantly lower. The white blood cell counts, serum albumin and C-reactive protein levels of patients were similar between the two groups. As for flora distribution, patients in the non-survival group were more associated with enterococci, non-fermenting bacteria and Candida albicans infection. The proportion of Enterobacteriaceae (38.7 vs. 39.3%, *P* = 0.915) and streptococci (2.7 vs. 9.3%, *P* = 0.057) were similar between the two groups (Table 2).

**Table 2:** Laboratory findings and organism distribution of ICU-IAIs patients.

	**Total**	**Survivors**	**Non-survivors**	**Hazard ratio**	**95% CI**	***p*-values**
	***N* = 476**	***N* = 400**	***N* = 76**			
**Laboratory findings**						
White blood cell (median, IQR), 10^9^/L	12.7 (7.8–17.8)	13.5 (8.4–18.2)	10.6 (4.3–14.5)	0.966	0.935–0.998	0.037
Hematocrit (median, IQR), %	30.0 (25.4, 35.4)	30.6 (26.2, 35.8)	26.9(23.0, 31.3)	0.925	0.892–0.959	<0.001
Platelet (median, IQR), 10^9^/L	157.0 (105.0, 224.5)	166.0 (110.0, 228.5)	131.0 (64.0, 182.5)	0.994	0.991–0.997	<0.001
Procalcitonin (median, IQR), ng/ml	7.0 (2.0–25.8)	6.7 (1.9–21.8)	11.7 (2.4–80.2)	1.013	1.007–1.019	<0.001
C-reactive protein (median, IQR), mg/L	141.7 (75.7–213.4)	144.5 (77.8–210.0)	135.7 (66.3–218.2)	0.999	0.996–1.002	0.452
Lactate level (median, IQR), mmol/L	1.9 (1.2–3.3)	1.8 (1.2 −2.8)	3.5 (1.8–6.4)	1.217	1.160–1.276	<0.001
Albumin (median, IQR), g/L	27.2 (24.5, 30.0)	27.2 (24.5, 29.7)	27.3 (24.7, 30.8)	0.980	0.943–1.018	0.293
pH (median, IQR)	7.34 (7.29, 7.39)	7.35 (7.30, 7.40)	7.32 (7.24, 7.39)	0.053	0.005–0.589	0.017
Creatinine (median, IQR), μmol/L	90.0 (64.0, 156.5)	84.5 (63.0, 139.5)	136.0 (78.0, 211.0)	1.001	0.999–1.002	0.240
Blood urine nitrogen (median, IQR), mmol/L	8.9 (5.8, 14.3)	8.3 (5.5, 13.3)	13.3 (7.8, 22.5)	1.038	1.020–1.056	<0.001
Total bilirubin (median, IQR), mmol/L	21.1 (11.4, 35.8)	17.9 (10.8, 30.0)	44.9 (22.8, 108.5)	1.004	1.003–1.005	<0.001
P/F ratio (median, IQR), mmHg	304.0 (229.5, 383.0)	313.5 (236.5, 390.5)	246.0 (187.0, 324.0)	0.995	0.993–0.998	<0.001
**Specific organism**, ***n*** **(%)**						
Non-fermentative bacterial	49/431 (11.4)	32/356 (9.0)	17/75 (22.7)	2.288	1.332–3.930	0.003
*Enterococcus*	119/431 (27.6)	86/356 (24.2)	33/75 (44.0)	2.122	1.344–3.349	0.001
Fungi	94/431 (21.8)	73/356 (20.5)	21/75 (28.0)	1.533	0.863–2.723	0.145

*ICU, intensive care unit; IAIs, Intra-Abdominal Infections; CI, confidence interval; IQR, interquartile range*.

*Laboratory test indicators are all from blood samples*.

Compared with the survival group, fewer patients in the non-survival group received surgery (68.4 vs. 89.8%, *P* < 0.001), but the surgical management was similar in both groups. The timing of surgical intervention and antibiotic treatment were similar in the two groups. Thirty-four patients were sent to the operating room from the ICU for surgery, but the time from diagnosis to surgery did not differ between the non-survivor and survivor groups (HR 0.994, 95% CI 0.973–1.015) (Supplementary Table 1). Patients in non-survival group had a higher rate of IAT failure tendency (*P* = 0.027). Besides, patients in the two groups received similar duration of mechanical ventilation, but the proportion of patients receiving continuous renal replacement therapy (CRRT), vasopressor drugs and glucocorticoid therapy in non-survival group was significantly higher than that of the survival group. In this study, norepinephrine was our preferred vasopressor drug, used in 62.4% (297/476) of patients, followed by dopamine, and in more severe cases which tend to have a poor prognosis, epinephrine was considered. The non-survival group received more positive fluid (*P* < 0.001) during different time periods in ICU (whether it was 24, 48, or 72 h). In addition, patients in the non-survival group had increased risk of postoperative complications, including anastomotic leakage, intestinal fistula, and abdominal abscess, while the incidence of incision infection was similar in the two groups (Table 3).

**Table 3:** Antibiotic, source control, and complications of IAIs patients in ICU.

	**Total**	**Survivors**	**Non-survivors**	**Hazard ratio**	**95% CI**	***p*-values**
	***N* = 476**	***N* = 400**	***N* = 76**			
**Treatment**						
Surgery	411 (86.3)	359 (89.8)	52 (68.4)	3.226	1.971–5.280	<0.001
Traditional laparotomy/Laparoscopic surgery (reference)	385 (93.7)/26 (6.3)	334 (93.3)/24 (6.7)	51 (96.2)/2 (3.77)	1.640	0.399–6.738	0.492
Time to surgery ^*^(median, IQR), hrs	4 (3, 7)	4 (3, 7)	4 (3, 8)	1.002	1.000–1.003	0.043
Time to IAT^#^ (median, IQR), hrs	2 (1–3)	2 (1–3)	2 (2, 8)	1.013	0.998–1.029	0.098
IAT failure	145 (30.5)	112 (28.0)	22 (43.4)	1.676	1.060–2.651	0.027
Mechanical ventilation	427 (89.7)	357 (89.3)	70 (92.1)	1.441	0.621–3.343	0.395
Vasopressor agents	307 (64.5)	242 (60.5)	65 (85.5)	3.256	1.698–6.168	<0.001
Norepinephrine	297 (62.4)	232 (58.0)	65 (85.5)	3.569	1.878–6.783	<0.001
Dopamine	42 (13.7)	32 (13.2)	10 (15.9)	1.139	0.579–2.243	0.7059
Epinephrine	11 (2.3)	4 (1.0)	7 (9.2)	4.332	1.988–9.442	<0.001
Dobutamine	26 (8.5)	17 (7.0)	9 (13.9)	1.701	0.832–3.479	0.1457
CRRT	94 (19.8)	51 (12.8)	43 (56.6)	5.526	3.498–8.729	<0.001
Glucocorticoid	218 (45.8)	160 (40.0)	58 (76.3)	4.197	2.462–7.155	<0.001
**Fluid balance**						
24 h (mean ± SD), 100 ml	16.0 ± 15.0	14.2 ± 13.5	26.2 ± 18.3	1.053	1.036–1.068	<0.001
48 h (mean ± SD), 100 ml	17.7 ± 23.0	15.0 ± 21.0	32.3 ± 27.5	1.037	1.026–1.049	<0.001
72 h (mean ± SD), 100 ml	14.3 ± 31.0	10.7 ± 29.4	30.4 ± 33.4	1.023	1.014–1.033	<0.001
**Local complications**, ***n*** **(%)**						
Anastomotic leakage	36/452 (8.0)	26/383 (6.8)	10/69 (14.5)	1.795	0.918–3.512	0.087
Intestinal fistula	36/452 (8.0)	24/383 (6.3)	12/69 (17.4)	2.233	1.197–4.163	0.012
Abdominal abscess	64/452 (14.2)	44/383 (11.5)	20/69 (29.0)	2.284	1.350–3.865	0.002
Incision infection	61/452 (13.5)	54/383 (14.1)	7/69 (10.1)	0.630	0.288–1.379	0.248

*IAIs, Intra-Abdominal Infections; ICU, Intensive Care Unit; CI, Confidence Interval; IQR, Interquartile Range; SD, Standard Deviation; IAT, Initial Antibiotic Therapy; CRRT, Continuous Renal Replacement Therapy*.

* *Time from clinical diagnosis to surgery*.

# *Time from clinical diagnosis to IAT*.

### Evaluation of Independent Risk Factors for 28-Day Mortality in ICU-IAIs

Variables selected in the univariate COX analysis were included in the multivariate COX regression analysis, and we found that underlying chronic diseases, high SOFA score, low hematocrit, and receiving more fluids within 72 h in ICU were independent risk factors for 28-day mortality (Table 4).

**Table 4:** Multivariate COX regression analysis of 28-day mortality.

**Variables**	**Adjusted hazard ratio**	**95% CI**	***p*-values**
Underlaying chronic disease	3.137	1.425–6.906	0.005
SOFA	1.285	1.160–1.424	<0.001
Hematocrit	0.910	0.861–0.960	<0.001
Fluid balance 72 h, 100 ml	1.028	1.015–1.041	<0.001

*CI, confidence interval; SOFA, Sequential Organ Failure Assessment*.

*Proportional-hazards assumption: chi 4.64, df 4, P =0.3265*.

### Comparison of Demographic Data, Clinical Characteristics, Treatment Status and Prognosis the First 4 Years (2011–2014) and the Last 4 Years (2015–2018)

ICU mortality, 28-day mortality and overall hospital mortality of the patients in the last 4 years (2015–2018) group were significantly lower than those of the patients in the first 4 years (2011–2014) group. While demographic data, clinical characteristics of the two groups were similar. The rates of surgical intervention, surgical management, and timing of surgical intervention in the two groups were similar, also the CRRT. And the rate of mechanical ventilation and glucocorticoid use in the last 4 years group was lower than that in the first 4 years group. In terms of antibiotic use, the last 4 years group was earlier and the failure rate of IAT was lower than that in the first 4 years group (26.9 vs. 35.5%). In addition, whether the positive fluid balance is 24, 48, or 72 h, patients in the last 4 years group received statistically less positive fluid balance than those in the first 4 years group at the timepoint of 24, 48, and 72 h after ICU admission (Table 5).

**Table 5:** Clinical characteristics of ICU-IAIs in the timepoint of 2011–2014 and 2015–2018.

	**2011–2014**	**2015–2018**	**Total**	**Statistics *t/z/χ^2^***	***p*-values**
	***n* = 197**	***n* = 279**	***n* = 476**		
Age (mean ± SD)	57.8 ± 16.9	58.0 ± 16.6	57.9 ± 16.7	−0.188	0.851
≥65 years, *n* (%)	78 (39.6)	114 (40.9)	192 (40.3)	0.077	0.781
Gender, male, *n* (%)	125 (63.5)	194 (69.5)	319 (67.0)	1.933	0.164
CA-IAIs/HA-IAIs, *n* (%)	95 (48.2)/102 (51.8)	114 (40.9)/165 (59.1)	209 (43.9)/267 (56.1)	2.542	0.111
Postoperative IAIs, *n* (%)	48 (24.4)	79 (28.3)	127 (26.7)	0.921	0.337
IAI type, *n* (%)				1.836	0.339
Diffuse	132 (67.0)	170 (60.9)	302 (63.5)	1.836	0.175
Localized	62 (31.5)	104 (37.3)	166 (34.9)	1.713	0.191
**Source of infection**, ***n*** **(%)**					
Stomach/duodenum	68 (34.5)	83 (29.8)	151 (31.7)	1.212	0.271
Intestine	94 (47.7)	153 (54.8)	247 (51.9)	2.347	0.126
Liver/gallbladder	19 (9.6)	16(5.7)	35 (7.4)	2.591	0.107
Pancreas	9 (4.6)	19 (6.8)	28 (5.9)	–	0.331
Pelvic cavity	2 (1.0)	2 (0.7)	4 (0.8)	–	1.000
Primary	5 (2.5)	4 (1.4)	9 (1.9)	–	0.499
Postoperative (gastrointestinal perforation)	0	2 (0.7)	2 (0.4)	–	0.514
Underlaying chronic disease, *n* (%)	102 (51.8)	145 (52.0)	247 (51.9)	0.002	0.967
COPD	7 (3.6)	19 (6.8)	26 (5.5)	2.372	0.124
Chronic heart failure	12 (6.1)	20 (7.2)	32 (6.7)	0.214	0.644
Metastatic cancer	38 (19.3)	65 (23.3)	103 (21.6)	1.094	0.296
Hematologic malignancy	2 (1.0)	8 (2.9)	10 (2.1)	–	0.207
Cirrhosis	19 (9.6)	16 (5.7)	35 (7.4)	2.591	0.107
Chronic renal failure	5 (2.5)	13 (4.7)	18 (3.8)	1.428	0.232
Immunosuppression	17 (8.6)	16 (5.7)	33 (6.9)	1.500	0.221
AIDS	1 (0.5)	0 (0.0)	1 (0.2)	–	0.414
Diabetes mellitus	29 (14.8)	32 (11.5)	61 (12.8)	1.138	0.286
APCHE-II (median, IQR)	15 (11, 20)	15 (11, 21)	15 (11, 20)	−0.652	0.515
SOFA (median, IQR)	5 (3, 8)	5 (3, 7)	5 (3, 7)	1.249	0.212
MPI (median, IQR)	26 (19, 31)	25 (20, 31)	25 (20, 31)	0.214	0.830
Recent antibiotic therapy, *n* (%)	48 (24.4)	77 (27.6)	125 (26.3)	0.623	0.430
**Clinical presentation**, ***n*** **(%)**					
Fever or hypothermia^*^	103 (52.3)	154 (55.2)	257 (54.0)	0.394	0.530
Tachypnea^#^	100 (50.8)	165 (59.1)	265 (55.7)	3.285	0.070
SIRS score ≥ 2	161 (81.7)	234 (83.9)	395 (83.0)	0.376	0.540
Septic shock	85 (43.2)	106 (38.0)	191 (40.1)	1.277	0.258
**Treatment**					
Surgery, *n* (%)	176 (89.3)	235 (84.2)	411 (86.3)	2.558	0.110
Traditional laparotomy/Laparoscopic surgery	162 (91.5)/15 (8.5)	223 (95.3)/11 (4.7)	385 (93.7)/26 (6.3)	2.422	0.120
Time to surgery (median, IQR), hrs	5 (3, 7)	4 (3, 7)	4 (3, 7)	1.184	0.237
Time to IAT (median, IQR), hrs	2 (1.5, 3)	2 (1, 3)	2 (1, 3)	2.142	0.032
IAT failure, *n* (%)	70 (35.5)	75 (26.9)	145 (30.5)	4.080	0.043
Mechanical ventilation, *n* (%)	184 (93.4)	243 (87.1)	427 (89.7)	4.970	0.026
Vasopressor agents, *n* (%)	135 (68.5)	172 (61.7)	307 (64.5)	2.386	0.122
CRRT	36 (18.3)	58 (20.8)	94 (19.8)	0.461	0.497
Glucocorticoid, *n* (%)	102 (51.8)	116 (41.6)	218 (45.8)	4.839	0.028
**Fluid balance**					
24 h (mean ± SD), ml	1,887.7 ± 1,415.0 (*n* = 194)	1,401.0 ± 1,523.5 (*n* = 278)	1,601.0 ± 1,497.6 (*n* = 472)	3.516	<0.001
48 h (mean ± SD), ml	2,485.8 ± 2,163.5 (*n* = 173)	1,281.9 ± 2,265.1 (*n* = 251)	1,773.1 ± 2,299.3 (*n* = 424)	5.478	<0.001
72 h (mean ± SD), ml	2,410.4 ± 2,653.3 (*n* = 56)	787.7 ± 3,214.9 (*n* = 87)	1,428.7 ± 3,104.7 (*n* = 143)	4.750	<0.001
**Prognosis**					
Mechanical ventilation time (median, IQR), *d*	2 (1, 5)	2 (1, 5)	2 (1, 5)	0.753	0.452
Length of ICU stay (median, IQR), *d*	4 (2, 9)	4 (3, 8)	4 (3, 8)	−0.632	0.527
Length of hospital stay (median, IQR), *d*	19 (12, 34)	20 (13, 36)	20 (12, 35)	−0.883	0.377
28-day mortality, *n* (%)	40 (20.3)	36 (12.9)	76 (16.0)	4.714	0.030
ICU mortality, *n* (%)	32 (16.2)	27 (9.7)	59 (12.4)	4.585	0.032
In-hospital mortality, *n* (%)	41 (20.8)	37 (13.3)	78 (16.4)	4.805	0.028

*ICU, Intensive Care Unit; IAIs, Intra-Abdominal Infections; SD, Standard Deviation; CI, Confidence Interval; IQR, Interquartile Range; CA, Community-acquired; HA, healthcare or hospital-associated; COPD, Chronic Obstructive Pulmonary Disease; AIDS, Acquired Immune Deficiency Syndrome; APACHE-II, Acute Physiology and Chronic Health Evaluation-II; SOFA, Sequential Organ Failure Assessment; MPI, Mannheim Peritonitis Index; SIRS, Systemic Inflammatory Response Syndrome; IAT, Initial Antibiotic Therapy; CRRT, Continuous Renal Replacement Therapy*.

* *T ≥ 38°C or T ≤ 36°C*.

# *Breath rath ≥ 22 bpm*.

## Discussion

In our retrospective study over 8 years, 51.9% of patients had underlying chronic diseases. The most common chronic diseases were metastatic cancer, diabetes, and immunosuppressive status. The median scores of APACHE-II and SOFA are 15[11,20] and 5[3,7], respectively. The ICU mortality, 28-day mortality, and overall hospital mortality of IAIs were 12.4, 16.0, and 16.4%, respectively. The 28-day mortality of patients with septic shock was 30.9%. And ICU mortality, hospital mortality, and 28-day mortality are lower than the EPIC-II study (3) and the AbSeS study (17), we considered that demographic data, treatment strategy adjustments, and heterogeneity of ICU may be the reasons for the differences, and the severity of disease may be milder than the AbSeS study (17), as the SOFA score were lower in our study.

There have been a large number of literature studies on the risk factors of IAIs, and many factors have been confirmed to be related to the mortality of IAIs patients. These risk factors are usually included clinical and physiological characteristics, infection severity, surgical intervention, microbial factors, antibiotic treatment status, and progression of diseases (15). Risk factors for mortality of IAIs include age, underlying chronic diseases, loss of consciousness, septic shock, admission to ICU, hypoalbuminemia, peak level of serum PCT and lactate concentration, high MPI score, high APACHE-II score, and high SOFA score, diffuse peritonitis, enterococci and fungi or drug-resistant bacteria infection (2, 9, 10, 18–21). In addition, hematological malignancies, liver cirrhosis, mechanical ventilation, renal replacement therapy and high Simplified Acute Physiology Score (SAPS) II scores have been confirmed to be independent risk factors of mortality for patients with ICU-IAIs (3). Enterococcal isolation is associated with mortality in elderly patients in ICU-IAIs (22), high APACHE-II, kidney injury, cardiovascular insufficiency, low hematocrit, and low body temperature are related to the death of patients with fecal peritonitis (11). Multivariate COX analysis identified underlying chronic diseases, high SOFA score, low hematocrit, and receiving more fluids within 72 h in ICU as independent risk factors for 28-day mortality in the present study. That is not surprising, underlying chronic diseases (18, 23–26) and SOFA score (27, 28) have been confirmed by numerous studies as a risk factor for mortality in patients with IAIs.

The relationship between hematocrit and patient prognosis is rarely mentioned (11, 29). In our study, the hematocrit at the time of ICU admission was related to the mortality of the patient, but the reason for this result is not clear to us. In severe IAIs, there are many factors that lead to a decrease in hematocrit. Loss of blood due to infection or surgery, and fluid resuscitation are common factors. If a patient is combined with septic shock, massive fluid resuscitation can lead to dilutional anemia, so that oxygen-carrying capacity decreases, which in turn causes insufficient tissue oxygen supply and ultimately affect the patient's prognosis. Whether infusion of red blood cells improves the prognosis of patients with ICU-IAIs remains to be further studied.

Timely removal of the source of infection, usually by surgery, is an important measure for the management of IAIs, which may affect the prognosis of patients. In our study, we found that the surgical intervention rate of the survival group (89.8%) was higher than that of the non-survival group (68.4%). The reason may be that patients in non-survival group had more underlying chronic diseases and were so critically ill that it is not suitable for them to receive surgical treatment. This indirectly indicates that the removal of infection is a crucial measure for the management of IAIs, which may affect the prognosis of patients, but surgery is not the only method, since surgical intervention was not an independent risk factor for mortality in the statistical analysis. Surprisingly, we found that the timing of surgical intervention (the time from the establishment of a clinical diagnosis to the operation) has no significant impact on the mortality of patients. This is different from the results of several earlier studies (9, 30), and is similar with the recent GenOSept study (11). The timing of surgical intervention in the GenOSept study was the time from the onset of peritonitis symptoms to the operation. In addition to surgical operations, the measures to control infections also included percutaneous drainage. For patients who are critically ill and intolerant for major surgery, percutaneous drainage of abscesses or effusions under ultrasound positioning is preferable to surgical intervention (14). It is too arbitrary to judge whether surgical intervention or infection source control is delayed simply judged by the time between onset and surgery, and sometimes the surgeon needs to decide the timing of surgery according to the patient's basic condition and clinical manifestations.

Early goal-directed therapy (EGDT) is a 6-h resuscitation program for patients with sepsis and has been included in the guidelines of surviving sepsis campaign (12, 31). Subsequent three studies consistently showed that EGDT did not reduce the mortality of patients with sepsis when compared with the conventional treatment program, in contrast, patients received more fluid resuscitation and cost more (32–34). Recent studies have shown that positive fluid balance is an independent risk factor for increased mortality in patients with sepsis (35). Another study also showed that prognosis of patients is related with fluid balance at any phase in the treatment course (36). Similarly, our study found that the 28-day mortality of ICU-IAIs patients was significantly related to the positive fluid balance within 72-h after ICU admission, which was detected to be an independent risk factor for mortality. Fluid resuscitation will promote tissue edema while restoring tissue perfusion, which is not helpful to improve tissue oxygen metabolism. This may be the reason why excessive fluid resuscitation increases the mortality of patients. In addition, fluid resuscitation on the basis of abdominal infection and surgical trauma can promote the exudation of tissues in the abdominal cavity, thereby causing abdominal hypertension or even causing abdominal compartment syndrome in severe cases, finally leading to insufficient perfusion of abdominal organs, for example, gastrointestinal tract, kidney, liver, etc. the uncontrolled vicious circle may cause sequential organ dysfunction, and increase the risk of death. In the comparison of the first 4 years (2011–2014) and the last 4 years (2015–2018), we found that the patients in the two periods were similar in terms of disease severity and underlying disease, but ICU mortality, hospital mortality and 28-day mortality of patients in the later period (2015–2018) were significantly lower than those in the previous period (2011–2014). Specifically, the daily fluid balance for the first 3 days was a risk factor for the mortality of patients. The lower positive fluid balance was significantly related to the reduced mortality, which further confirmed that the positive fluid balance is a risk factor for increased mortality in patients with sepsis (35).

The standard antibiotic treatment of IAIs should cover gram-negative enterobacteria, aerobic streptococci and intestinal anaerobes. In critically ill patients, pathogens are more complicated and assessment is more difficult. Additional antibiotics may be needed to cover uncommon drug resistant or opportunistic pathogens (14, 37). When this principle is not followed or the initial antibiotic treatment cannot effectively cover the pathogens, the mortality rate of IAIs is significantly increased (26, 38–40). Our study found that failure of initial antibiotic therapy (IAT) is a risk factor associated with mortality, but we have not been able to confirm that failure of initial antibiotic therapy is an independent risk factor for mortality in ICU-IAIs.

This study has some limitations. First of all, this is a single-center retrospective observational study. The patients with IAIs in the study may not fully represent the situation in other regions. Secondly, ICU-IAIs refer to IAIs admitted to the ICU. There are both severe IAIs and IAIs combined with other critical illnesses. When referring to the conclusions of this study, we need to recognize that the actual situation of our included data. Third, it is difficult to distinguish whether the isolated microorganism is pathogenic or contaminated, as this is a retrospective study, so the definition of IAT failure may be biased. Fourth, fluid overload maybe to bring about the problem of intra-abdominal hypertension. During the study, we found 35 patients with high risk factors underwent intra-abdominal pressure monitoring. These patients were selected, and there was bias, and these small amounts of data are difficult to help us to determine the relationship between intra-abdominal pressure changes and fluid load. Finally, a considerable number of patients were transferred from other hospitals. The time onset of IAIs is sometimes difficult to define. Therefore, it is difficult to determine the time from the onset of symptoms to the operation. In this way, we did not analyze the relationship between surgery delay (defined as the time from the onset of symptoms to the surgery over 24 h) and 28-day mortality.

## Conclusion

The 28-day mortality of ICU-IAIs was 16.0%. Underlying chronic diseases, high SOFA score, low hematocrit, and receiving more fluids within 72 h in ICU were independent risk factors for 28-day mortality. Comparing the first 4 years (2011–2014) and the last 4 years (2015–2018), the early use of antibiotics, the optimization of IAT strategies, and the restriction of positive fluid balance are related to the decline in mortality of IAIs in the last 4 years (2015–2018).

## Data Availability Statement

The original contributions presented in the study are included in the article/Supplementary Material, further inquiries can be directed to the corresponding author/s.

## Ethics Statement

The studies involving human participants were reviewed and approved by the Ethical Committee of Nanfang Hospital, Southern Medical University (No. NFEC-2019-162). Written informed consent for participation was not required for this study in accordance with the national legislation and the institutional requirements.

## Author Contributions

XL analyzed the data and wrote the article. LL collected the data. SO helped to analyzed the data and reviewed the tables. ZZ reviewed and modified the article. ZC designed the study. All authors contributed to the article and approved the submitted version.

## Funding

This work was funded by the Natural Science Foundation of China (81871604) and the Natural Science Foundation of Guangdong Province (2017A030313590 and 2016A030313561).

## Conflict of Interest

The authors declare that the research was conducted in the absence of any commercial or financial relationships that could be construed as a potential conflict of interest.

## Publisher's Note

All claims expressed in this article are solely those of the authors and do not necessarily represent those of their affiliated organizations, or those of the publisher, the editors and the reviewers. Any product that may be evaluated in this article, or claim that may be made by its manufacturer, is not guaranteed or endorsed by the publisher.
