# Supplementary material for: Risk Factors for Mortality in Abdominal Infection Patients in ICU: A Retrospective Study From 2011 to 2018
Source: Front Med (Lausanne). 2022 Feb 25;9:839284. doi: 10.3389/fmed.2022.839284 (PMC8916228; doi:10.3389/fmed.2022.839284)
Supplement: Supplementary file 1 [file Table_1.docx]

STable 1. The time between diagnosis and surgical treatment for patients in the ward.

|  | **Total**  **N=34** | **Survivors**  **N=27** | **Non-Survivors**  **N=7** | **Hazard Ratio** | **95% CI** | **p-values** |
| --- | --- | --- | --- | --- | --- | --- |
| Time to surgery *(median, IQR), hrs | 4 (5, 10) | 4 (6, 12) | 4 (4, 4) | 0.994 | 0.973-1.015 | 0.563 |
